# Supplementary material for: Breast Tumor Characterization Using [18F]FDG-PET/CT Imaging Combined with Data Preprocessing and Radiomics
Source: Cancers (Basel). 2021 Mar 12;13(6):1249. doi: 10.3390/cancers13061249 (PMC8000810; doi:10.3390/cancers13061249)
Supplement: Supplementary file 1 [file cancers-13-01249-s001.pdf]

# Breast tumor characterization using [$^{18}$F]FDG-PET/CT imaging combined with data preprocessing and radiomics

Denis Krajnc[1], Laszlo Papp[1], Thomas S. Nakuz[2], Heinrich F. Magometschnigg[3], Marko Grahovac[2,4], Clemens P. Spielvogel[2,4], Boglarka Ecsedi[1], Zsuzsanna Bago-Horvath[5], Alexander Haug[2,4], Georgios Karanikas[2], Thomas Beyer[1], Marcus Hacker[2], Thomas H Helbich[3], Katja Pinker[3,6]

[1]QIMP Team, Center for Medical Physics and Biomedical Engineering, Medical University of Vienna, Vienna, Austria

[2]Medical University of Vienna, Department of Biomedical Imaging and Image-guided Therapy, Division of Nuclear Medicine, Vienna, Austria

[3]Medical University of Vienna, Department of Biomedical Imaging and Image-guided Therapy, Division of Molecular and Gender Imaging, Vienna, Austria

[4]Medical University of Vienna, Christian Doppler Laboratory for Applied Metabolomics, Vienna, Austria

[5] Department of Pathology, Medical University of Vienna, Vienna, Austria;

[6]Department of Radiology, Breast Imaging Service, Memorial Sloan Kettering Cancer Center, New York, NY, USA

Correspondence:

Thomas Beyer, PhD, MBA
QIMP Team, Center for Medical Physics and Biomedical Engineering, Medical University of Vienna, Vienna, Austria
Medical University Vienna
Währinger Gürtel 18-20
1090 Vienna, Austria
thomas.beyer@meduniwien.ac.at

## SUPPLEMENTAL MATERIALS

**Supplemental Table S1**: Imaging Biomarker Standardization Initiative (IBSI) reporting structure of the study. The information presented herein is based on the IBSI guidelines [1]

| | |
|---|---|
| **Patient** | |
| Volume of Interest | PET/CT positive lesions in breast |
| Patient Preparation | Patients were required to fast for at least 5 h before injection of (200–350) MBq $^{18}$F-FDG based on body weight with blood glucose level <150 mg/dL (8.3 mmol/L). |
| Radiotracer | [$^{18}$F]FDG $^{18}$F-Fluorodeoxyglucose |
| **Acquisition and Reconstruction** | |
| Protocol | A dedicated breast PET/CT scan was performed over one PET bed position with the patient in the prone position. |
| Scanner type | Siemens Biograph 64 TruePoint® PET/CT |
| [$^{18}$F]FDG | - 200–350 MBq injected<br>- < 150 mg/dL (8.3 mmol/L) blood glucose level<br>- 5 min acquisition time<br>- 60 min uptake time<br>- 605 mm transaxial FOV<br>- Iterative TrueX reconstruction, 4 iterations per 21 subsets<br>- 168 × 168 axial matrix size<br>- 3.6 × 3.6 × 5 mm voxel size |
| **Image Co-registration** | |
| Software | Hermes Hybrid 3D ver 4.0 |
| Co-registration step | Automated as of DICOM coordinate parameters |
| **Data conversion** | |
| Step 1 (all images) | Initial voxel values determined by transforming the DICOM raw voxel values with the DICOM tags Rescale Scope (0028|1053) and Rescale Intercept (0028|1052). |
| Step 2 (PET) | Initial voxel values transformed to tumor-to-background ratio (TBR) by dividing all voxel values with the mean of the reference region drawn as a 4 × 4 × 4 cuboid VOI in the mediastinum region in each patient. |
| **Delineation** | |
| Software | Hermes Hybrid 3D ver 4.0 |
| VOI definition | Standard semi-automated iso-count 3D VOI tools |

| | |
|---|---|
| Number of experts | 1 + 1 (1 nuclear medicine expert participated in independent delineations, followed by 1 senior nuclear medicine specialist cross-validation and if necessary, modification of first-round results) and 1 + 1 (1 breast imaging expert participated in independent delineations, followed by 1 breast imaging specialist cross-validation and if necessary, modification of first-round results) |
| Reference image | PET |
| **Image / VOI interpolation** | |
| Method | Kriging interpolation in 3D, including nearest neighbors in distance of voxel size main diagonal [2] |
| Grid | Align by voxel center |
| Extrapolation beyond original image | Neighbor distance search calculated as original voxel size main diagonal + epsilon. Missing value: image minimum |
| Voxel dimensions | 1.0 mm and 4.0 mm uniform voxel sizes as of [3] |
| Partially masked voxels (VOI) | If more than half of original voxel area included |
| **Discretization** | |
| Method | Fixed bin width, variable number of bins |
| Bin width | 0.01 and 5 [3] |
| **Image biomarker computation / Parameters** | |
| Biomarker set | - Intensity-based statistical features (6 per image): Minimum intensity, Maximum intensity, Mean intensity, Intensity variance, Intensity range (Maximum – Minimum), Intensity sum<br>- Intensity histogram features (6 per image): Discretised intensity uniformity, Discretised intensity entropy, (Excess) discretised intensity kurtosis, Mean discretised intensity, Discretised intensity skewness, Discretised intensity variance<br>- GLCM features (18 per image): Angular second moment, Autocorrelation, Cluster prominence, Cluster shade, Contrast, Correlation, Difference entropy, Difference variance, Dissimilarity, Joint entropy, Joint maximum, Joint variance, Inverse difference, Inverse difference moment, Sum average, Sum entropy, Sum variance, Information correlation 1<br>- GLSZM features (11 per image): Gray level non-uniformity, High gray level zone emphasis, Large zone high grey level emphasis, Large zone low grey level emphasis, Large zone emphasis, Low grey level zone emphasis, Small zone high grey level emphasis, Small zone low grey level emphasis, Small zone emphasis, Zone size non-uniformity, Zone percentage |

| | |
|---|---|
| | - Morphological features (4): Volume (voxel counting), Compactness 1, Spherical disproportion, Area<br>- NGTDM features (5 per image): Coarseness, Contrast, Busyness, Complexity, Strength |
| Custom set | - Fusion features (14 per image): Angular second moment, Auto correlation, Cluster prominence, Cluster shade, Contrast, Correlation, Dissimilarity, Entropy, Information correlation, Inverse difference, Inverse difference moment, Maximum probability, Normalized mutual information, Sum of squares variance<br>Fusion features are generated from a 2D joint histogram [4, 5] for which overlapping voxel values coming from images A and B are determined within the given VOI mask. The generated joint histogram is afterwards handled in the same way as it was a GLCM. |
| Software | MUW radiomics engine 2.0 [3]. Software availability upon reasonable request from the corresponding author. |
| Distance weighting | No |
| CM symmetry | Symmetric |
| CM / ZM distance | Chebyshev distance 1 |
| CM / ZM aggregation | 3D, full-merging |
| Exclusion criteria | VOIs with less than 64 voxels were excluded from the analysis |

## Machine learning predictive models

Five random forest (RF) algorithms [6] with different hyperparameter configuration were employed in an ensemble learning scheme. The final model decision was obtained by averaging across the five predictive models. (See Supplemental Table S2).

**Supplemental Table S2**: Algorithms settings of the 5 RF models employed in the ensemble learning scheme [6]. KDE = Kernel Density Estimation [7].

| Parameter | RF-1 | RF-2 | RF-3 | RF-4 | RF-5 |
|---:|---|---|---|---|---|
| Number of trees | 100 | 400 | 300 | 300 | 200 |
| Quality metric | | | gain | | |
| Max depth | 5 | 10 | 10 | 15 | 15 |
| Min samples at leaf | | | 5 | | |
| Feature selection | | | Random | | |
| KDE attributes per split | 10 | 15 | 20 | 25 | 25 |
| Random features | 10 | 6 | 4 | 4 | 4 |
| Number selected trees | | | 10 | | |
| Bagging method | | | equalized | | |
| Bag fraction | | | 1.0 | | |

## Data preparation pipelines across all machine learning predictive models

In order to perform data preparation steps on the training data, a pipeline was established following the logic where firstly, the dataset is cleansed from outliers by utilizing the Isolation Forest algorithm [8], High feature dimensionality was considered and handled by applying Sequential Forward Selection (SFS) [9] feature selection algorithm, followed by removal of noise and borderline samples with Tomek Link [10]. Furthermore, the dataset was balanced by utilizing advanced oversampling methods such as Synthetic Minority Oversampling Technique (SMOTE) [11].

**Supplemental Table S3:** Data preparation pipelines across all machine learning predictive models.

| Model | Algorithms pipeline |
|---|---|
| **Breast cancer detection (malignant vs benign)** | Isolation forest -> SFS -> Tomek Links -> SMOTE |
| **ER** | |
| **PR** | |
| **HER2** | |
| **Ki-67** | |
| **Triple negative** | |
| **Luminal A/B** | |

SFS = Sequential Forward Selection; SMOTE = Synthetic Minority Oversampling Technique; ER = estrogen; HER2 = Human Epidermal growth Receptor 2; PR = progesterone

## Best performing machine learning predictive models over sham data

**Supplemental Table S4:** Machine learning results of best performing models (per reference label) over sham data. Confusion matrix values are presented as percentages (%), while area under the curve (AUC) values are presented as ratios.

| Model | ACC | SENS | SPEC | NPV | PPV | AUC |
|---|---|---|---|---|---|---|
| **Breast cancer detection (malignant vs benign)** | 47 | 46 | 49 | 47 | 47 | 0.48 |
| **ER** | 54 | 67 | 41 | 55 | 53 | 0.52 |
| **PR** | 49 | 70 | 27 | 48 | 49 | 0.48 |
| **HER2** | 50 | 17 | 84 | 50 | 52 | 0.48 |
| **Ki-67** | 52 | 50 | 55 | 52 | 53 | 0.51 |
| **Triple negative** | 59 | 47 | 72 | 57 | 62 | 0.59 |
| **Luminal A/B** | 51 | 15 | 88 | 50 | 55 | 0.47 |

ACC = Accuracy; AUC = Area under the receiver operator characteristic curve; ER = Estrogen; HER2 = Human Epidermal growth Receptor 2; NPV = Negative Predictive Value; PPV = Positive Predictive Value; PR = Progesterone; SENS = Sensitivity; SPEC = Specificity.

## Conventional positron emission tomography (PET)-based correlation analysis

**Supplemental Table S5**: Conventional positron emission tomography (PET)-based correlation analysis for malignancy, estrogen (ER), progesterone (PR), human epidermal growth receptor 2 (HER2), Ki-67 protein, triple negative, and luminal A/B status, expressed in *P*-values.

| **Model** | | $SUV_{mean}$ | | $SUV_{min}$ | | $SUV_{max}$ | | $SUV_{peak}$ | | $SUV_{TLG}$ | |
|---|---|---|---|---|---|---|---|---|---|---|---|
| | | mean ± σ | *P*-value | mean ± σ | 0.21226 | mean ± σ | *P*-value | mean ± σ | *P*-value | mean ± σ | *P*-value |
| **Malignancy** | malignant | 2.98 ± 0.68 | 0.00004 | 1.82 ± 0.57 | 0.00026 | 5.81 ± 1.37 | 0.00021 | 3.81 ± 0.96 | 0.00145 | 44.71 ± 19.12 | 0.21226 |
| | benign | 1.67 ± 1.79 | | 0.75349 | | 2.48 ± 5.25 | | 1.81 ± 3.68 | | 10.88 ± 162.98 | |
| **ER** | + | 2.80 ± 1.59 | 0.00509 | 1.85 ± 0.79 | 0.79181 | 5.14 ± 4.17 | 0.00016 | 3.44 ± 2.92 | 0.00289 | 42.75 ± 184.45 | 0.75349 |
| | − | 4.17 ± 2.48 | | 0.43446 | | 10.58 ± 8.42 | | 6.52 ± 6.38 | | 57.43 ± 103.19 | |
| **PR** | + | 2.80 ± 1.44 | 0.03739 | 1.87 ± 0.78 | 0.7778 | 5.08 ± 3.64 | 0.0032 | 3.40 ± 2.59 | 0.0185 | 36.91 ± 176.59 | 0.43446 |
| | − | 3.67 ± 2.53 | | 0.21124 | | 8.73 ± 8.24 | | 5.48 ± 5.96 | | 67.78 ± 162.46 | |
| **Ki-67** | + | 3.14 ± 1.76 | 0.00269 | 1.85 ± 0.72 | 0.03718 | 6.45 ± 5.20 | 0.00286 | 4.16 ± 3.61 | 0.00521 | 55.90 ± 197.27 | 0.21124 |
| | − | 1.98 ± 0.77 | | 0.39311 | | 3.10 ± 1.69 | | 2.00 ± 0.98 | | 4.66 ± 4.38 | |
| **HER2** | + | 2.79 ± 1.48 | 0.53045 | 1.76 ± 0.65 | 0.5554 | 5.38 ± 3.92 | 0.54183 | 3.27 ± 2.40 | 0.37348 | 16.75 ± 27.83 | 0.39311 |
| | − | 3.07 ± 1.93 | | 0.47311 | | 6.20 ± 5.89 | | 4.12 ± 4.22 | | 52.67 ± 193.90 | |
| **Triple negative** | Yes | 4.94 ± 2.68 | 0.00019 | 1.99 ± 0.72 | 0.5459 | 13.36 ± 9.17 | 0.000001 | 8.26 ± 7.25 | 0.00006 | 80.45 ± 121.89 | 0.47311 |
| | No | 2.78 ± 1.55 | | 0.61971 | | 5.13 ± 4.07 | | 3.41 ± 2.85 | | 40.45 ± 137.34 | |
| **Luminal A/B** | A | 2.96 ± 1.58 | 0.70784 | 1.80 ± 0.71 | 0.81099 | 5.84 ± 4.29 | 0.80613 | 3.61 ± 3.37 | 0.85397 | 17.90 ± 32.67 | 0.61971 |
| | B | 2.79 ± 1.60 | | *P*-value | | 5.51 ± 4.84 | | 3.44 ± 2.63 | | 41.11 ± 177.88 | |

SUV = standard uptake value;

## Performance comparison of machine learning predictive models and standard uptake value (SUV)-based predictive models

**Supplemental Table S6:** Holomics-based vs standard uptake value (SUV)-based ML performance comparison across all predictive models.

| Model | | SENS | SPEC | NPV | PPV | ACC |
|---|---|---|---|---|---|---|
| **Breast cancer detection (malignant vs benign)** | ML-based | 80 | 78 | 79 | 78 | 80 |
| | SUV-based | 71 | 44 | 61 | 58 | 56 |
| **ER** | ML-based | 82 | 56 | 76 | 69 | 65 |
| | SUV-based | 86 | 23 | 62 | 55 | 53 |
| **PR** | ML-based | 78 | 35 | 61 | 56 | 54 |
| | SUV-based | 77 | 37 | 62 | 57 | 55 |
| **HER2** | ML-based | 17 | 84 | 50 | 50 | 51 |
| | SUV-based | 2 | 80 | 45 | 41 | 09 |
| **Ki-67** | ML-based | 62 | 68 | 64 | 65 | 66 |
| | SUV-based | 78 | 48 | 68 | 63 | 60 |
| **Triple negative** | ML-based | 85 | 78 | 84 | 79 | 82 |
| | SUV-based | 51 | 82 | 63 | 67 | 74 |
| **Luminal A/B** | ML-based | 16 | 89 | 51 | 53 | 59 |
| | SUV-based | 14 | 82 | 49 | 48 | 44 |

ACC = Accuracy; ER = estrogen; HER2 = Human Epidermal growth Receptor 2; NPV = Negative Predictive Value; PPV = Positive Predictive Value; PR = progesterone; SENS = Sensitivity; SPEC = Specificity.

Predictive model performance is expressed in percentages (%).

## References

1. Zwanenburg A, Vallières M, Abdalah MA, Aerts HJWL, Andrearczyk V, Apte A, et al. The Image Biomarker Standardization Initiative: Standardized Quantitative Radiomics for High-Throughput Image-based Phenotyping. Radiology. 2020;295:328-38. doi:10.1148/radiol.2020191145.
2. Stytz MR, Parrott RW. Using kriging for 3d medical imaging. Computerized Medical Imaging and Graphics. 1993;17:421-42. doi:10.1016/0895-6111(93)90059-V.
3. Papp L, Rausch I, Grahovac M, Hacker M, Beyer T. Optimized feature extraction for radiomics analysis of 18 F-FDG-PET imaging. Journal of Nuclear Medicine. 2018:jnumed.118.217612-jnumed.118. doi:10.2967/jnumed.118.217612.
4. Sun S, Guo C. Medical Image Registration by Maximizing a Hybrid Normalized Mutual Information. IEEE. p. 964-7.
5. Oubel E, Beaumont H, Iannessi A. Mutual information-based feature selection for radiomics. In: Zhang J, Cook TS, editors. p. 97890L-L.
6. Papp L, Spielvogel CP, Grubmüller B, Grahovac M, Krajnc D, Ecsedi B, et al. Supervised machine learning enables non-invasive lesion characterization in primary prostate cancer with [68Ga]Ga-PSMA-11 PET/MRI. European Journal of Nuclear Medicine and Molecular Imaging. 2020. doi:10.1007/s00259-020-05140-y.
7. Geng X, Hu G. Unsupervised feature selection by kernel density estimation in wavelet-based spike sorting. Biomedical Signal Processing and Control. 2012;7:112-7. doi:10.1016/j.bspc.2011.03.002.
8. Liu FT, Ting KM, Zhou ZH. Isolation forest. Proceedings - IEEE International Conference on Data Mining, ICDM. 2008:413-22. doi:10.1109/ICDM.2008.17.
9. Marcano-Cede??o A, Quintanilla-Dom??nguez J, Cortina-Januchs MG, Andina D. Feature selection using Sequential Forward Selection and classification applying Artificial Metaplasticity Neural Network. IECON Proceedings (Industrial Electronics Conference). 2010:2845-50. doi:10.1109/IECON.2010.5675075.
10. Elhassan T, Aljurf M, F A-M, Shoukri M. Classification of Imbalance Data Using Tomek Link ( T-Link ) Combined with Random Under-sampling ( RUS ) as A Data Reduction Method Sampling-based Methods Basic Sampling Methods. Journal of Informatics and Data Mining. 2016;1:1-12.
11. Chawla NVBKWHLOKWP. SMOTE: Synthetic Minority Over-Sampling Technique. Journal of Artificial Intelligence Research. 2002;16:321-57. doi:10.1613/jair.953.
